# A novel greedy heuristic‐based approach to intraoperative planning for permanent prostate brachytherapy

**DOI:** 10.1120/jacmp.v16i1.5144

**Published:** 2015-01-08

**Authors:** Bin Liang, Fugen Zhou, Bo Liu, Junjie Wang, Yong Xu

**Affiliations:** ^1^ Image Processing Center Beihang University Beijing China; ^2^ The Center of Oncology Peking University Third Hospital Beijing China; ^3^ Department of Urology the General Hospital of PLA Beijing China

**Keywords:** greedy heuristic, intraoperative planning, prostate brachytherapy

## Abstract

This paper presents a greedy heuristic‐based double iteration and rectification (DIR) approach to intraoperative planning for permanent prostate brachytherapy. The DIR approach adopts a greedy seed selection (GSS) procedure to obtain a preliminary plan. In this process, the potential seeds are evaluated according to their ability to irradiate target while spare organs at risk (OARs), and their impact on dosimetric homogeneity within target volume. A flexible termination condition is developed for the GSS procedure, which guarantees sufficient dose within target volume while avoids overdosing the OARs. The preliminary treatment plan generated by the GSS procedure is further refined by the double iteration (DI) and rectification procedure. The DI procedure removes the needles containing only one seed (single seed) and implements the GSS procedure again to get a temporary plan. The DI procedure terminates until the needles number of the temporary plan does not decrease. This process is guided by constantly removing undesired part rather than imposing extra constrains. Following the DI procedure, the rectification procedure attempts to replace the remaining single seeds with the acceptable ones within the existing needles. The change of dosimetric distribution (DD) after the replacement is evaluated to determine whether to accept or to withdraw the replacement. Experimental results demonstrate that the treatment plans obtained by the DIR approach caters to all clinical considerations. Compared with currently available methods, DIR approach is faster, more reliable, and more suitable for intraoperative treatment planning in the operation room.

PACS number: 87

## I. INTRODUCTION

Transrectal ultrasound (TRUS)‐guided permanent prostate brachytherapy^(1)^ has become a standard cure for localized prostate cancer.^(2)^ As the energy emitted by commonly used seeds attenuates rapidly along with the distance, the dosimetric distribution (DD) within regions of interest (ROIs) greatly depends on the positions of implanted seeds. Hence treatment plan, which determines the positions of implanted seeds, directly affects operation outcome.

The treatment plan can be designed several days prior to (preplanning) or just before immediate execution of the plan (intraoperative planning).^(3)^ Compared with preplanning, intraoperative planning avoids the need for two separate TRUS procedures and a reproducible patient positioning. It is also reported that intraoperative planning‐based implants achieve better biological therapeutic effect and lower possibility of morbidity.^4^, ^5^, ^6^

Currently available optimization methods can be classified into stochastic, deterministic, and heuristic approaches.^(7)^ Stochastic approaches are proposed by Pouliot et al.,^(8)^ Yu and Schell,^(9)^ Yu et al.,^(10)^ and Yang et al.^(11)^ Based on either simulated annealing (SA) or genetic algorithm (GA), these approaches can generate a feasible treatment plan quickly, and can be used for intraoperative planning.

Deterministic approaches were reported by Lee et al.,^(12)^ Lee and Zaider, ^(13)^ and D'Souza et al.^(14)^ Treatment planning is modeled as a mixed‐integer programming (MIP) problem. And the problem is solved by branch‐and‐bound (BB) approach. In practice, the goal is often set to search a feasible solution that satisfies all constraints, not necessarily the optimal solution. Otherwise, the optimization process would take several days to be completed.^(15)^

Heuristic approaches were reported by Yoo et al.^15^, ^16^ and Chaswal et al.^17^, ^18^ For each step, the potential seeds are evaluated according to their ability to irradiate target volume while spare organs at risk (OARs), and the optimal seed is selected until sufficient dose is delivered to target volume. In this process, in order to prevent selected seed from congregating, an isodose surface‐based constraint is used to exclude the potential seeds which are close to the selected ones. In order to limit the number of used needles, Yoo and colleagues confine the search space within existing needles when needles number reaches the predetermined threshold. Chaswal and colleagues define a penalty function for the seed requiring adding a new needle.

The approach presented in this paper is also based on greedy heuristic. In our previous work, we developed an improved seed evaluation criterion.^(19)^ We continued the work and developed an adaptive termination condition. The termination condition guarantees sufficient target coverage while avoids overdosing OARs. Another innovation of this approach is the DI and rectification procedure, which reduces the puncture needles without degrading the quality of treatment plan. Experiments show the algorithm can generate a satisfactory treatment plan in about 30 sec. This approach provides an alternative option for intraoperative treatment planning.

## II. MATERIALS AND METHODS

**Figure 1:**
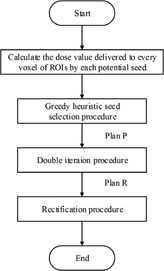
The flowchart of the DIR approach.

The flowchart of this approach is shown in Fig. 1. First, the dose value delivered to every voxel of prostate, margin, urethra, and rectum volume (PMUR) by each potential seed is calculated. Then the greedy seed selection (GSS) procedure is implemented, which yields a preliminary plan (plan P). Then plan P is refined by the double iteration (DI) procedure. The refined plan (plan R) is further modified by the rectification procedure.

### A. Dose calculation

The set of potential seeds is denoted as $S_{p}$, and the set of selected seeds is denoted as $S_{s}$. At the beginning stage, $S_{p}$ contains all potential seeds, and $S_{s}$ is empty. The dose value ($D_{\text{ij}}$) delivered to every voxel of PMUR by each potential seed is calculated according to Eq. (1):

(1) $$D_{ij}=D(r)=\frac{S_{k}\lambda }{r^{2}}g(r)\Phi_{an}\;(i\in S_{p},\;j\in PMUR)$$

where *i* is the potential seed in $S_{p}$ and *j* is the voxel of PMUR; *r* is the distance between the positions of *i* and *j*. Detailed description of other parameters can be found in Nath et al.^(20)^ The mean dose delivered to the organs of PMUR $(\bar{P_{i}},\bar{U},\bar{R}$ and $\bar{M})$ by each potential seed is:

(2) $$\begin{array}{l} \bar{P_{i}}=\frac{\sum_{j=1}^{N_{p}}D_{ij}}{N_{p}}(i\in S_{p},\;j\in \text{Prostate})\;\bar{U_{i}}=\frac{\sum_{j=1}^{N_{u}}D_{ij}}{N_{p}}\;(i\in S_{p},\;j\in \text{Urethea})\\ \bar{R_{i}}=\frac{\sum_{j=1}^{N_{r}}D_{ij}}{N_{r}}(i\in S_{p},\;j\in \text{Rectum})\;\bar{M_{i}}=\frac{\sum_{j=1}^{N_{m}}D_{ij}}{N_{m}}\;(i\in S_{p},\;j\in \text{Margin}) \end{array}$$

where $N_{p},N_{u},N_{r}$, and $N_{m}$ are the voxel number of prostate, urethra, margin, and rectum volume, respectively.

Given a set of selected seeds $S_{s}$, the dose value delivered to PMUR is:

(3) $$D_{j}^{'}=\sum_{i=1}^{N_{s}}D_{ij}\;(i\in S_{s},\;j\in PMUR)$$

where $N_{s}$ is the number of the selected seeds.

### B. The GSS procedure

**Figure 2:**
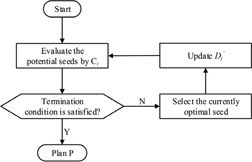
The flowchart of the GSS procedure.

The flowchart of the GSS procedure is shown in Fig. 2. The GSS procedure selects (transferred from $S_{p}$ to $S_{s}$) one seed at a time until termination condition is satisfied. For each time, the potential seeds are assessed by the evaluation criterion ($C_{i}$). The seed with minimum value is considered as the currently optimal seed ($o,o\;S_{p}$). If the termination condition is not reached, o is selected, and ${D_{j}}^{\prime }(j\;\varepsilon \;\text{PMUR})$ is updated by adding the dose value delivered to PMUR by $\left(D_{\text{oj}},j\;\varepsilon \;\text{PMUR}\right)$ Otherwise, the GSS procedure terminates and the seeds in $S_{s}$ constructs plan P.

(4) $$D_{j}^{'}=\sum_{i=1}^{N_{s}}D_{ij}+D_{oj}\;(i\in S_{s},\;j\in PMUR,\;o\in S_{p})$$

#### B.1 Seed evaluation criterion

It has been demonstrated that the mean dose delivered to PMUR by the potential seed reflects its ability to irradiate these organs.^(15)^ As the objective is to deliver sufficient and uniform dose to target volume while spare $\text{OARs},C_{i}$ is defined as:

(5) $$C_{i}=\frac{\bar{U_{i}}+\bar{R_{i}}+\bar{M_{i}}}{\bar{P_{i}}}\times Dev_{i}\;i\in S_{p}$$

where ${\text{Dev}}_{i}$ is the deviation of dose value within prostate volume after the seed *i* is selected

(6) $$Dev_{i}=\frac{\sqrt{\sum_{j=1}^{N_{p}}{\left(D_{j}^{'}+D_{ij}-\bar{D^{'}}+\bar{P_{i}}\right)}^{2}}}{N_{p}}(i\in S_{p},\;j\in \text{Prostate})$$

where $\bar{D^{\prime }}$ is the mean value of the current dose value within target volume

(7) $$\bar{D^{'}}=\frac{\sum_{j=1}^{N_{p}}D_{j}^{'}}{N_{p}}\;(j\in \text{Prostate})$$

#### B.2 Termination condition

The termination condition of the GSS procedure is defined based on the target coverage and OAR damage. The target coverage ($P_{100}$) is the percentage of target volume within which the dose value is equal to or greater than the prescription dose ($D_{p}$). The OAR damage is assessed by the percentage of urethra ($U_{120}$) and rectum ($R_{80}$) volume within which the dose value is equal to or greater than 120% and 80% $D_{p}$, respectively. $P_{100},U_{120}$, and $R_{80}$ are calculated as follows:

(8) $$P_{100}=\frac{\sum_{j=1}^{N_{p}}\Theta (D_{j}^{'}-D_{p})}{N_{p}}\;j\in \text{Prostate}$$

(9) $$U_{120}=\frac{\sum_{j=1}^{N_{u}}\Theta (D_{j}^{'}-120\%\times D_{p})}{N_{u}}\;j\in \text{Urethra}$$

(10) $$R_{80}=\frac{\sum_{j=1}^{N_{r}}\Theta (D_{j}^{'}-80\%\times D_{p})}{N_{r}}\;j\in \text{Rectum}$$

where Θ(x) is the Heaviside function. Through experiments we found that when $P_{100}$ reaches nearly 90%, the target region receiving lower dose is closely adjacent to urethra and rectum. In this case, the gain of $P_{100}$ is at the expense of rapid increase of $U_{120}$ and $R_{80}$.^(14)^ In order to guarantee sufficient target coverage while avoiding overdosing urethra and rectum volume, the termination condition is defined as follows:

Stage 1: when $P_{100}$ is below 95% (this threshold value is determined according to the criterion recommended by AAPM^3^, ^21^), the currently optimal seed ($o,o\;\varepsilon \;S_{P}$) is selected.
Stage 2: when $P_{100}$ is greater than 95%, o is selected and the increase of $P_{100}$ ($\Delta P_{100}$), $U_{120}$($\Delta U_{120}$), and $R_{80}$ ($\Delta R_{80}$) is calculated:

(11) $$\Delta P_{100}=\frac{\sum_{j=1}^{N_{p}}\Theta (D_{j}^{'}+D_{oj}-D_{p})}{N_{r}}-P_{100}\;(j\in \text{Prostate},\;o\in S_{p})$$

(12) $$\Delta U_{120}=\frac{\sum_{j=1}^{N_{u}}\Theta (D_{j}^{'}+D_{oj}-120\%\times D_{p})}{N_{u}}-U_{120}\;(j\in \text{Urethra},\;o\in S_{p})$$

(13) $$\Delta R_{80}=\frac{\sum_{j=1}^{N_{r}}\Theta (D_{j}^{'}+D_{oj}-80\%\times D_{p})}{N_{r}}-R_{80}\;(j\in \text{Rectum},\;o\in S_{p})$$

Whether to keep or withdraw o is determined by the ratio (R) of the sum of $\Delta U_{120}$ and $\Delta R_{80}$ to $\Delta P_{100}$:

(14) $$R=\frac{\Delta U_{120}+\Delta R_{80}}{\Delta P_{100}}$$

A greater value of R indicates that o will cause relatively severe OAR damage but marginal target coverage increase. In this case, o is removed and the GSS procedure terminates. Otherwise, o is kept and the GSS procedure continues.

In our work, the threshold value of $R(T_{R})$ is fixed to 10, which means one percent increase of $P_{100}$ should cause no greater than ten percentage of the total increase of $U_{120}$ and $R_{80}$. The threshold value is suitable for the ten tested patient cases used in this paper. It could be adjusted to cater to different priorities.

### C. Double iteration procedure

When the GSS procedure terminates, the selected seeds ($S_{s}$) construct plan P. Plan P contains considerable needles carrying only one seed (single seed). The DI procedure aims to refine plan P by reducing these single seeds.

**Figure 3:**
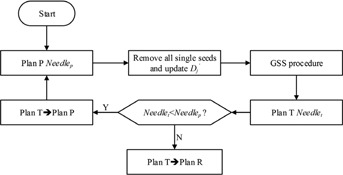
The flowchart of the DI procedure.

The flowchart is shown in Fig. 3. Plan P is the input of the DI procedure. First, all the single seeds are removed, and ${D_{j}}^{\prime }$ is updated by subtracting the corresponding dose value. Then the GSS procedure is implemented, which yields a temporary plan (plan T). The needles number of plan $T({\text{Needle}}_{t})$ is compared with plan $P({\text{Needle}}_{p})$. If plan T uses fewer needles than plan $P({\text{Needle}}_{t}<{\text{Needle}}_{p})$, it is used as the input of the next iteration procedure. Otherwise, the DI procedure terminates and plan T is the output.

For each step of the GSS procedure, the optimal seed for the current DD is selected. During DI procedure, the removed single seeds may not be optimal for the updated DD, and these seeds may not be selected. If the selected seed is in the existing needles — in other words, does not require adding a new needle — it is kept and remain valid.

**Figure 4:**
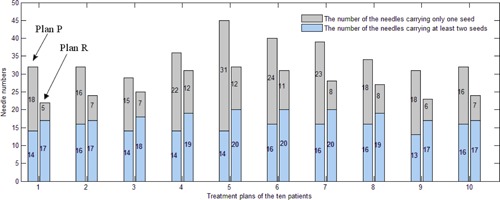
The needles numbers of the treatment plans before and after the DI procedure. The gray bar is the number of needles carrying one seed. The blue bar is the number of the needles carrying at least two seeds.

### D. Rectification procedure

The rectification procedure aims to further refine plan R by replacing the remaining single seeds with the acceptable seeds in the existing needles. The rectification procedure attempts to replace one single seed at a time; it terminates when all single seeds are processed. For each time, the replacement is evaluated by the change of $P_{100},U_{120},R_{80}$ and dose nonuniformity ratio (DNR) within target volume. DNR is defined as the ratio of $P_{150}$ to $P_{100}$. $P_{150}$ is the percentage of target volume within which the dose value is equal to or greater than 150% $D_{p}$:^(22)^

(15) $$DNR=\frac{P_{150}}{P_{100}}$$

(16) $$P_{150}=\frac{\sum_{j=1}^{N_{p}}\Theta (D_{j}^{'}-150\%\times D_{p})}{N_{p}}j\in \text{Prostate}$$

**Figure 5:**
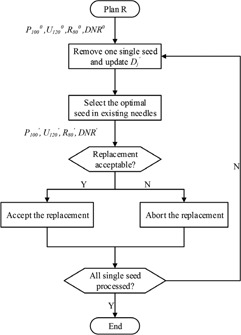
The flowchart for the rectification procedure.

The flowchart is shown in Fig. 5. One single seed is removed, then ${D_{j}}^{\prime }$ is updated by subtracting the corresponding dose value. The potential seeds in the existing needles are evaluated by $C_{i}$, and the optimal seed is selected. The total increase of $U_{120}$, R80, and DNR (AURD) is calculated:

(17) $$\Delta URD=U_{120}^{'}-U_{120}^{0}+R_{80}^{'}-R_{80}^{0}+DNR^{'}-DNR^{0}$$

where ${P_{100}}^{0},{U_{120}}^{0},{R_{80}}^{0},{DNR}^{0},{P_{100}}^{\prime },{U_{120}}^{\prime },{R_{80}}^{\prime }$ and ${\text{DNR}}^{\prime }$ are the values of $P_{100},U_{120},R_{80}$, and DNR before and after the replacement, respectively. The criteria used to determine whether to keep or withdraw the replacement is defined as:

1. ${P_{100}}^{\prime }$ should be greater than 95%.
2. ΔURD should be below the threshold ($T_{\text{urd}}$).

The value of $T_{\text{urd}}$ is determined by pondering between the concerns about OAR damage, DNR, and the number of puncture needles. In our work, the value of $T_{\text{urd}}$ is fixed to 0.1, which is suitable for the tested patient data. It could be adjusted to sustain different practical variations.

## III. RESULTS

Ten patient data were used to test the efficiency of the DIR approach. Among currently available approaches, the heuristic approach is significantly faster and the deterministic approach can generate more satisfactory treatment plan. So we implemented the heuristic approach reported by Yoo et al. (YH)^15^, ^16^ and the improved deterministic (BB) approach^(14)^ using the same patient data for comparison.

The detailed description of the patient data is listed in Table 1. The margin region is the 3 mm layer outside the PTV of prostate. The voxel grid is set to $1\times 1\times 5\;{\text{mm}}^{3}$. And the voxel numbers of the PMUR are listed in Table 1. The positions of seeds and needles are the determined by the 5 mm grid template. For the three approaches, only the seeds and needles within target volume are considered as the potential seeds and needles. The numbers of potential seeds ($N_{\text{ps}}$) and needles ($N_{\text{pn}}$) are listed in Table 1. The same type of radioactive seed, $0.4\;\text{mCi}\;{}^{125}I$, is used for the three approaches, and $D_{p}$ is all set to 145 Gy. All the three approaches require one to calculate $D_{\text{ij}}$ at the beginning stage, and the dose calculation time is also listed in Table 1.

Both DIR and YH approach are implemented with C++ language. And the BB approach is implemented with the CPLEX MIP solver of the General Algebraic Modeling System (GAMS).^(23)^ All the three algorithms are executed on the computer with AMD Athlon(tm)II×4 (3.00G Hz) processor. Note: we limit the computational time of BB approach to two hrs; otherwise, it may take a rather long time to complete.

**Table 1:** Detail description of patient data: the volume (cc) and voxel numbers of prostate, margin, urethra, and rectum; the numbers of the potential needles and seeds; and the dose calculation time (sec)

	*Prostate*		*Margin*		*Urethra*		*Rectum*				
*Patient*	*Vol*.	$N_{p}$	*Vol*.	$N_{m}$	*Vol*.	$N_{u}$	*Vol*.	$N_{r}$	$N_{\text{ps}}$	$N_{\text{pn}}$	*Time*
1	31.8	6879	11.6	1338	0.6	127	4.7	1072	270	45	0.56
2	34.2	6702	11.6	1168	1.0	211	5.6	1150	281	51	0.61
3	28.8	5680	9.7	932	1.0	198	6.0	1255	251	53	0.48
4	36.5	7059	12.9	1302	0.7	142	5.5	1149	300	50	0.66
5	41.4	8093	12.8	1385	1.1	229	4.4	927	354	69	0.81
6	37.2	8039	12.4	1407	0.6	133	4.4	1042	315	66	0.73
7	47.1	10226	14.9	1730	0.9	208	5.3	1261	398	62	1.11
8	35.3	7503	12.1	1369	0.6	126	3.6	858	296	52	0.62
9	32.0	6074	11.1	1098	0.8	173	8.0	1688	256	50	0.53
10	32.5	6140	10.6	1078	0.9	195	7.6	1576	245	51	0.57

### A. The YH approach

The YH approach is also based on greedy heuristic. The YH approach adopts an isodose‐based strategy to constrain the search space by eliminating the potential seeds which are closed to the selected ones. And the search space is further confined within the existing needles when the number of used needles reaches the threshold (determined by the size of target volume). If there are no available seeds while the target coverage is not sufficient, all the selected seeds are removed, the constraint parameters are adjusted, and the procedure starts from scratch. This process is later referred as one iteration.

The seed evaluation criterion is defined as:

(18) $$C_{i}^{'}=\frac{\alpha \times \bar{U_{i}}+\beta \times \bar{R_{i}}+\gamma \times \bar{M_{i}}}{\bar{P_{i}}}i\in S_{p}$$

where α,β, and γ are the weighting factors, and other parameters are the same as Eq. (5). It is reported that the weighting factors reflect the concern about the protection of OARs.^(16)^ For example, if one intends to reduce the dose value delivered to urethra volume, the value of a should be increased.

At first, we set the value of α,β, and γ to 1 and found that the dose value delivered to rectum volume is acceptable, but the dose value delivered to urethra is much higher than acceptable level. So we increased the value of α in order to achieve better protection of urethra volume. The critical parameters of the generated treatment plans for Patients 1 and 2 are shown in Table 2.

Although the value of α is increased, the value of $U_{120}$ does not change as expected. The reason may be the strategy used to constrain the search space. In the process of seed selection, the search space is determined by the selected ones. If the value of α is adjusted, the selected seed of each step will be different, which results in different search space for the next step. For each step, the YH approach always selects the optimal seed from the current search space without considering the dose value within OARs, and it terminates when sufficient target coverage is achieved. Therefore, the generated treatment plans may be uncontrollable.

For the ten tested patient data, the value of α is set to 1–6 to generate six treatment plans. And the most satisfactory treatment plan is chosen for comparison.

**Table 2:** The critical parameters of the treatment plans for Patients 1 and 2 obtained by using different values of α

*Patient*	α	*Needle*	*Seed*	$P_{100}\;(\%)$	*DNR*	$U_{120}\;(\%)$	$R_{80}\;(\%)$
1	1	23	54	98.6	0.518	95.1	11.9
	2	19	57	99.3	0.605	96.2	22.3
	3	21	54	99.3	0.487	4.0	23.8
	4	22	52	99.2	0.425	23.9	20.0
	5	21	57	99.6	0.576	28.2	41.0
	6	22	53	99.4	0.437	46.4	29.0
2	1	18	60	98.9	0.702	85.1	34.3
	2	23	59	99.7	0.513	9.8	25.9
	3	20	60	99.3	0.581	43.1	24.5
	4	20	59	99.2	0.567	49.2	25.5
	5	21	59	98.9	0.513	29.4	14.9
	6	21	59	99.2	0.505	10.1	28.0

### B. The BB approach

The BB approach models the potential seed as a binary variable ($B_{i}$), and the dose value delivered to prostate, urethra, and rectum volume calculated as:

(19) $$D_{j}=\sum_{i=1}^{N_{seed}}B_{i}\times D_{ij}\;j\in \text{Prostate}\;\cup \;\text{Urethra}\;\cup \;\text{Rectum}\;B_{i}=0,\;or\;1$$

where $N_{\text{seed}}$ is the number of potential seeds.

We adopt the model reported by D'Souza et al.^(14)^ The objective function and the constraints of this model are:

(20) $$\begin{array}{l} \text{min}\;obj=\alpha \times \sum_{j\in \text{prostate}}(D_{p}-D_{j})\times \Theta (D_{p}-D_{j})+\beta \times \sum_{j\in \text{Urethra}}\left(D_{j}-120\%\times D_{p})\times \Theta (D_{j}-120\%\times D_{p}\right)\\ \;\;+\gamma \times \sum_{j\in rectum}(D_{j}-80\%\times D_{p})\times \Theta (D_{j}-80\%\times D_{p})+\delta \times N_{needle} \end{array}$$

(21) $$s.t.\;D_{j}\leq 150\%\times D_{p}\;j\in \text{Urethra}$$

(22) $$D_{j}\leq D_{p}\;j\in \text{Rectum}$$

In Eq. (20), α,β,γ, and δ are the weighting factors, $N_{\text{needle}}$ is the number of used needles. In D'Souza et al.,^(14)^ the values of these weighting factors are determined through experiments. In our work, the values of α,β,γ, and δ are determined by the principle reported by Lee et al.^(12)^

### C. Experiment results

The primary criteria for treatment planning recommended by AAPM(^3)^ are listed in Table 3. $D_{90},D_{10}$ and $D_{2\text{cc}}$ are defined as the minimum dose in the “hottest” certain percentage (90% and 10%) or certain size (2cc) of the volume. For target volume, if $P_{100}$ is greater than 95%, $D_{90}$ will be surely greater than $D_{p}$.^(3)^ The two criteria are equivalent to each other.

Apart from these parameters, we also compared $\text{DNR},U_{120}$, and $R_{80}$ of the generated plans. These critical parameters, along with the numbers of needles, seeds, iterations, and computational time, are all listed in Table 4. The computational time does not include the time consumed by dose calculation. The value of α of the YH approach is also listed in Table 4.

The treatment plans generated by the three approaches all cater to the planning criteria, which indicates all the three algorithms could generate feasible treatment plans.

**Table 3:** The criteria for the DD within target volume and OARs

	*Prostate*	*Urethra*	*Rectum*
Criterion	$D_{90}\geq D_{p}=145\;\text{Gy}(P_{100}\geq 95\%)$	$D_{10}<150\%D_{p}=217.5\;\text{Gy}$	$D_{2\text{cc}}<D_{p}=145\;\text{Gy}$

**Table 4:** The critical parameters of the treatment plans obtained by the three approaches

	*Configuration*					*Prostate*			*Urethra*		*Rectum*	
*Patient*	*Method*	*Needle*	*Seed*	*Time (sec)*	*Iteration*	$P_{100}\;(\%)$	$D_{90}\;(\text{Gy})$	*DNR*	$D_{10}\;(\text{Gy})$	$U_{120}\;(\%)$	$D_{2\text{cc}}\;(\text{Gy})$	$R_{80}\;(\%)$
1	YH(α=3)	21	54	59.19	1474	99.3	169.7	0.487	169.7	4.0	100.1	23.8
	BB	18	54	7200	NA	98.0	165.3	0.492	171.1	3.3	88.5	2.5
	DIR	19	50	12.15	3	98.6	168.2	0.328	171.1	2.8	92.8	16.9
2	YH(α=2)	23	59	81.75	1765	99.7	172.6	0.513	172.6	9.8	107.3	25.9
	BB	20	57	7200	NA	97.7	165.3	0.490	169.7	1.6	88.5	2.6
	DIR	23	52	11.28	2	97.4	163.9	0.356	175.5	14.8	91.4	10.9
3	YH(α=5)	24	52	88.13	2714	99.6	172.6	0.486	166.8	1.8	105.9	24.5
	BB	19	50	7200	NA	98.4	161.0	0.362	166.8	1.9	81.2	2.0
	DIR	20	48	10.33	4	97.9	162.4	0.360	174.0	12.9	87.0	9.3
4	YH(α=4)	21	63	56.27	1083	99.2	172.6	0.563	185.6	55.9	105.9	24.0
	BB	23	58	7200	NA	98.3	162.4	0.394	172.6	8.5	84.1	0.7
	DIR	27	54	13.21	3	97.6	169.7	0.382	187.1	51.5	85.6	6.9
5	YH(α=4)	29	67	195.53	2918	99.5	166.8	0.492	163.9	0.5	104.4	24.9
	BB	25	68	7200	NA	97.9	166.8	0.505	165.3	0.0	88.5	2.6
	DIR	27	60	30.36	3	97.3	166.8	0.346	172.6	5.6	97.2	15.6
6	YH(α=6)	26	60	111.34	2025	99.2	163.9	0.484	165.3	5.2	88.5	8.0
	BB	23	60	7200	NA	98.7	162.5	0.405	165.4	1.1	71.0	2.7
	DIR	31	56	32.79	5	98.0	163.9	0.287	166.8	0.0	79.8	4.3
7	YH(α=2)	27	71	147.75	1609	98.7	171.1	0.516	175.5	17.6	76.9	0.1
	BB	18	72	7200	NA	99.2	165.3	0.487	166.8	0.5	82.7	0.5
	DIR	23	64	46.50	4	96.4	162.4	0.337	172.6	8.9	68.2	0.0
8	YH(α=6)	24	58	122.55	2754	99.1	168.2	0.522	214.6	28.3	98.6	27.9
	BB	36	57	7200	NA	98.6	159.5	0.390	168.2	1.9	85.6	4.0
	DIR	23	51	16.94	3	97.3	161.0	0.316	172.6	5.6	100.1	35.1
9	YH(α=4)	17	54	6.01	152	99.5	171.1	0.499	178.4	18.3	78.3	1.6
	BB	15	56	7200	NA	99.7	172.6	0.512	171.1	4.6	79.8	0.6
	DIR	19	50	10.95	2	97.0	161.0	0.343	171.1	7.3	76.9	1.0
10	YH(α=6)	21	54	59.19	1474	98.3	167.0	0.496	169.7	14.0	110.1	26.8
	BB	18	54	7200	NA	98.2	163.4	0.461	173.2	6.8	84.6	4.5
	DIR	19	50	12.15	3	98.5	164.5	0.370	174.5	5.6	98.2	18.0

#### C.1 Comparison of $U_{120},R_{80}$, and DNR

6, 7 demonstrate $U_{120}$ and $R_{80}$ values of these plans, respectively. The BB approach has the best performance on the category of OAR protection. The DIR approach outperforms the YH approach on this category. Although the three approaches have different performance, the dose value delivered to OARs is kept within acceptable level of toxicity.

Because of the flexible termination condition, the DIR approach achieves better OAR protection at the expense of one or two percentage lower target coverage. However, the lowest target coverage of the DIR plans is 96.4%, and the $D_{90}$ is greater than $D_{p}$ which is still sufficient and feasible.

Figure 8 shows DNR values of these plans. The DNR values of the DIR plans are significantly lower than that of the BB and YH plans. This indicates the DD within target volume of DIR plans is more uniform.

**Figure 6:**
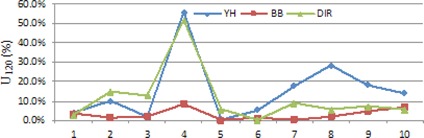
$U_{120}$ values of the treatment plans for the ten patients.

**Figure 7:**
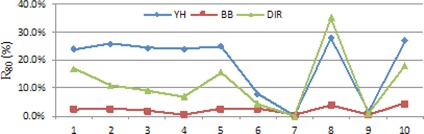
$R_{80}$ values of the treatment plans for the ten patients.

**Figure 8:**
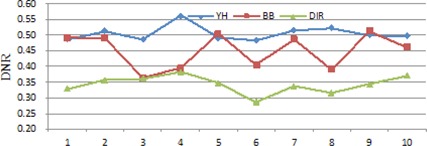
DNR values of the treatment plans for the ten patients.

#### C.2 Comparison of the computational time

**Figure 9:**
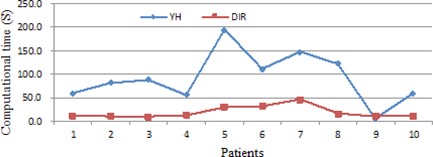
Computational time of the DIR and YH approach.

Figure 9 shows the computational time of the DIR and YH approaches. The computational time of the DIR approach is proportional to the size of target volume. For the largest prostate (47.1 cc), the computational time is 46.5 sec. The YH approach involves considerable iterations, which is to remove all selected seeds and start from scratch; thus it takes longer time to obtain the final solution.

#### C.3 Comparison of the numbers of used needles and seeds

**Figure 10:**
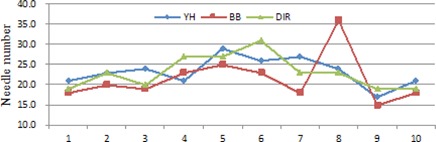
Needles numbers of the treatment plans for the ten patients.

Figure 10 shows the needles numbers of these plans. The DIR approach and the YH approach have similar performance on this category. In general, the BB plans use slightly fewer needles than the DIR and YH plans. However, the treatment plan for patient 8 uses far more needles than the plans obtained with the other two approaches. This indicates that the set of weighting factors are not suitable for this patient.

**Figure 11:**
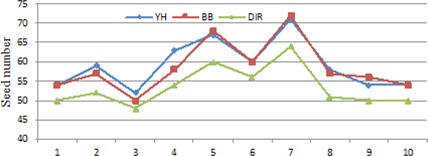
Seed numbers of the treatment plans for the ten patients.

Figure 11 shows the seed numbers of these plans. The DIR plans use slightly fewer seeds than the BB and YH plans. This coincides with the fact that the DIR plans achieve better homogeneity within target volume.

#### C.4 Seed and dosimetric distribution on each slice

**Figure 12:**
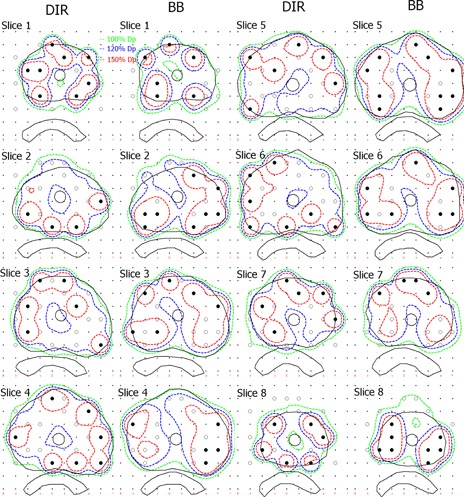
Isodose line and seed distribution of the DIR and BB plans for Patient 1. The isodose line is depicted in different colors and the selected seeds are marked by solid dots.

Figure 12 shows the isodose line and the selected seed of the DIR and BB plans on each slice. The $100\%\;D_{p}$ isodose line demonstrates that both plans achieve satisfactory target coverage. The $120\%\;D_{p}$ isodose line of the DIR plan has better conformity than the BB plan. And the $150\%\;D_{p}$ isodose line demonstrates that the higher dose region of the DIR plan is significantly lower than that of the BB plan.

## IV. DISCUSSION & CONCLUSIONS

Compared with the YH approach, the DIR approach yields better treatment plan in a shorter time. The improvements result from the improved seed evaluation criterion, the flexible termination condition, and the DI and rectification procedure.

Compared with the BB approach, the DIR plans achieve much better homogeneity. The BB approach protects OARs more effectively. However, the most obvious obstacle of using the BB approach for intraoperative planning is the computational time. In D'Souza et al.,^(14)^ the approach is implemented using the 2D data. The computational time is 20–45 min, and it would be much longer if 3D data are used. In Yoo et al.,^(16)^ the computational time is limited to 2 hrs. The BB approach fails to generate treatment plans for two out of ten patients. Although, the hardware and the algorithm have been greatly improved since then, we assert it is very difficult to get a feasible treatment plan using the BB approach within several minutes.

Another feature of the BB approach is that the quality of generated treatment plan is greatly relied on the values of the weighting factors. In practice, the values need to be adjusted to get a satisfactory plan. As the case of Patient 8, the value of δ needs to be increased to reduce the number of used needles. Meanwhile, other parameters (such as $P_{100},U_{120}$, and $R_{80}$) also need to be considered in this process. Given the computational time, this process will be rather time‐consuming.

In summary, the DIR approach presented in this paper is able to generate a feasible treatment plan for prostate brachytherapy quickly. The generated treatment plan achieves satisfactory dosimetric distribution. And the DIR approach is faster and more robust than the currently available approaches. Thus, the DIR approach is potential for intraoperative planning.

## ACKNOWLEDGMENTS

This work is supported by National Natural Science Foundation of China (61171005).
